# Isolation and characterization of native probiotics for fish farming

**DOI:** 10.1186/s12866-018-1260-2

**Published:** 2018-09-20

**Authors:** Konrad M. Wanka, Thilo Damerau, Benjamin Costas, Angela Krueger, Carsten Schulz, Sven Wuertz

**Affiliations:** 1 Department of Ecophysiology and Aquaculture, Leibniz-Institute of Freshwater Ecology and Inland Fisheries (IGB), Müggelseedamm 310, 12587 Berlin, Germany; 2 Gesellschaft für Marine Aquakultur mbH (GMA), Hafentörn 3, 25761 Büsum, Germany; 3 Albrecht Daniel Thaer-Institute of Agricultural and Horticultural Sciences, Humboldt University Berlin, Unter den Linden 6, 10099 Berlin, Germany; 4 Centro Interdisciplinar de Investigação Marinha e Ambiental (CIIMAR), Novo Edifício do Terminal de Cruzeiros do Porto de Leixões, Avenida Norton de Matos S/N, 4450-208 Matosinhos, Portugal; 5 Instituto de Ciências Biomédicas Abel Salazar (ICBAS-UP), Universidade do Porto, Rua de Jorge Viterbo Ferreira 228, 4050-313 Porto, Portugal; 6 Institute for Animal Breeding and Husbandry, Christian-Albrechts-University Kiel, Hermann-Rodewald-Straße 6, Kiel, Germany

**Keywords:** Aquaculture, Probiotic supplementation, Saponin metabolization, PUFA, *Psychrobacter*, *Acinetobacter*, Oral administration, Diet preparation, *Tenacibaculum maritimum*

## Abstract

**Background:**

Innovations in fish nutrition act as drivers for the sustainable development of the rapidly expanding aquaculture sector. Probiotic dietary supplements are able to improve health and nutrition of livestock, but respective bacteria have mainly been isolated from terrestrial, warm-blooded hosts, limiting an efficient application in fish. Native probiotics adapted to the gastrointestinal tract of the respective fish species will establish within the original host more efficiently.

**Results:**

Here, 248 autochthonous isolates were cultured from the digestive system of three temperate flatfish species. Upon 16S rRNA gene sequencing of 195 isolates, 89.7% (*n* = 175) Gram-negatives belonging to the Alpha- (1.0%), Beta- (4.1%) and Gammaproteobacteria (84.6%) were identified. Candidate probiotics were further characterized using in vitro assays addressing 1) inhibition of pathogens, 2) degradation of plant derived anti-nutrient (saponin) and 3) the content of essential fatty acids (FA) and their precursors. Twelve isolates revealed an inhibition towards the common fish pathogen *Tenacibaculum maritimum,* seven were able to metabolize saponin as sole carbon and energy source and two isolates 012 *Psychrobacter* sp. and 047 *Paracoccus* sp. revealed remarkably high contents of docosahexaenoic acid (DHA) and eicosapentaenoic acid (EPA). Furthermore, a rapid and cost-effective method to coat feed pellets revealed high viability of the supplemented probiotics over 54 d of storage at 4°C.

**Conclusions:**

Here, a strategy for the isolation and characterization of native probiotic candidates is presented that can easily be adapted to other farmed fish species. The simple coating procedure assures viability of probiotics and can thus be applied for the evaluation of probiotic candidates in the future.

## Background

Among animal food sources, fish are considered particularly important, providing high quality protein and essential nutrients such as polyunsaturated fatty acids (PUFA), minerals and vitamins. Undoubtedly, in the context of stagnating fisheries landings combined with increasing per capita consumption of fish worldwide, the demand for fish can only be met by a sustainable development of the aquaculture industry, where resources are by far more efficiently used [1, 2]. Here, limited feedstuff – most importantly fishmeal and fish oil – challenges future expansion. Thus, innovations in fish nutrition may act as drivers for the development of this industrial sector. In this context, functional diets that provide benefits by targeting specific physiological mechanisms to improve the health and immune status or optimize growth and feed conversion have a huge potential. Among dietary supplements, probiotics have been widely assessed in functional diets of terrestrial livestock but have an expandable potential in fish nutrition.

In fish nutrition, the mentioned limitations in fishmeal and the rapid growth of the aquaculture sector led to numerous research efforts in exploring replacements for fishmeal as protein source. Several studies focusing on protein-enriched agricultural by-products such as press cakes from plant oil production or residuals from starch production [3–5]. If plant-based proteins are used, anti-nutrients are a major constraint [6–8] affecting the performance of the farmed species, including reduced growth, feed conversion as well as manifold pathological alterations [3–5]. Often, plant derived ingredients such as soy or other legumes cause intestinal enteritis via detrimental secondary metabolites like saponin [9–12]. For example, most fish species do not tolerate less than 50% fishmeal replacement by soybean protein, commonly assigned to the anti-nutritional effects of saponin [11].

In 1905, Metchnikoff was the first to point out the positive role of bacteria in milk and yoghurt products for human health. Probiotics have therefore been defined as bioactive, living microbial food/feed additives, which have a positive influence on the digestion and, moreover, the microflora of the gastrointestinal tract (GIT) in general [13–15], improving nutrition as well as disease resistance. Among those beneficial effects, probiotics have been reported to improve a) disease resistance & immunity [16–18], b) nutrition & feed utilization [7, 19], c) reproduction & development [20–23], e) gut morphology and functioning [24, 25] as well as f) counteracting spinal curvature [26, 27].

In the past, aquaculture research has focused on well-known probiotic strains derived from terrestrial hosts, ignoring fundamental differences in the physiology of cultured species – mammals or birds versus fish – as well as those differences related to the environment where the respective microbial communities actually evolved - aquatic versus terrestrial environment. Therefore, probiotic bacteria isolated from the respective fish host are expected to perform better in their natural habitat than those derived from terrestrial hosts [28]. Thus, it is not surprising that in most cases, effects observed during the actual application, vanish rather fast if administration is terminated [29, 30]. Only recently, native probiotic candidates have been explored, mostly in classical fish feeding trials focusing on the evaluation of growth and feed conversion as well as immune modulation [31–34]. Such feeding trials are costly, time consuming and only feasible with a few candidates. Thus, fast in vitro screening strategies have to be established, allowing the selection of the most promising isolates. Ideally, in vitro screening would allow the identification of beneficial effects and reduce the risk of negative impacts. Furthermore, such research may provide new insights on the biology and ecology of autochthonous bacteria and improve the knowledge of microbial host interactions [35–37].

Used as prophylaxis or therapeutic treatment, probiotics act via competitive exclusion of the respective pathogen or directly inhibiting its propagation [38, 39]. Therefore, probiotics represent an alternative in disease prophylaxis and treatment, particularly in the context of emerging antibiotic resistance reported from aquatic environments in general [40–43] as well as from aquaculture sites [44, 45].

Nutritional effects reported upon probiotic administration include a better supply of nutrients such as essential fatty acids and their precursors [14]. PUFA namely EPA and DHA provide health benefits for the consumer, including optimal development of the nervous system during early ontogeny, reduced risk of abnormal heart rhythms, heart failure or strokes as well as anti-inflammatory effects. Consequently, probiotics may improve product quality, e.g., enrichment of essential fatty acids.

In this study, we evaluated candidate probiotics native to closely related flatfish species using an in vitro screening approach. Therefore, morphologically different colonies were isolated and characterized based on 16S rRNA sequence information. Consequently, isolates that could be stored as cryo-culture were evaluated using specific in vitro assays on 1) the antagonism towards three relevant fish pathogens *Tenacibaculum maritimum*, *Edwardsiella tarda*, *Listonella anguillarum*, 2) the synthesis of essential fatty acids EPA, DHA and precursors such as octadecatrienoic acid (ALA), octadecadienoic acid (LA), eicosatetraenoic acid (ETA) and 3) the metabolization of saponin as an example of a plant derived anti-nutrient. Finally, a cost-effective method for the supplementation of probiotics to fish feed was evaluated, assessing the viability of the respective bacteria upon top coating during storage.

## Results

### Identification of isolated bacteria

Initially, 248 morphologically distinct bacterial isolates from the intestinal tract of healthy individuals of three flatfish species were collected and archived in cryo-vials at − 80 °C. Fifty-three isolates died or could not be recovered from cryo-cultures. Sequence analysis of the partial 16S rRNA gene (907 bp) of 195 isolates revealed that these isolates were assigned to 21 distinct bacterial genera including 10.3% Gram-positive of seven genera and 89.7% Gram-negative strains of 14 genera. Besides 1.0% Alpha- and 4.1% Betaproteobacteria the majority of 84.6% Gram-negative isolates were Gammaproteobacteria (twelve genera). Gram-positive isolates comprised 6.7% Actinobacteria (five genera) and 3.6% Firmicutes including the genera *Bacillus* and *Staphylococcus* (Table 1). At least 25 isolates (14.0%) were known to be opportunistic or pathogenic, most of which were members of the Gammaproteobacteria class (Table 1). Several isolates were closely related to bacterial strains that had already been subjected to previous probiotic studies.

**Table 1:** Molecular identification of isolates based on partial 16S rRNA gene sequence information

**Phylogenetic classification**					**Consensus**	**BLAST analysis**		**No. of**	**New entry to**	**Pathogeni-**
**Phylum**	**Class**	**Family**	**Closest relative**	**Cluster**	**length [bp]**	**Accession no.**	**Similarity [%]**	**isolates**	**NCBI GenBank** ^**a**^	**city**
Firmicutes	Bacilli	Bacillaceae	*Bacillus cereus*		860	CP023245.1	96	1	MH512875	none
		Staphylococcaceae	*Staphylococcus saprophyticus*		887	CP022093.1	100	1	–	presumed
			*Staphylococcus* sp.	1	858	MF417799.1	100	5	–	none
Actinobacteria	Actinobacteria	Dietziaceae	*Dietzia* sp.		840	KP713427.1	100	1	–	none
		Microbacteriaceae	*Agrococcus* sp.		830	HM222687.1	99	1	MH512857	none
			*Microbacter. esteraromaticum*		859	KJ812392.1	100	1	–	none
			*Microbacter. phyllosphaerae*		857	LT223598.1	100	3	–	none
			*Microbacterium* sp.	1	830	AM403655.1	100	3	–	none
		Micrococcaceae	*Arthrobacter* sp.	1	842	FR693359.1	97	1	MH512858	none
			*Arthrobacter* sp.	2	839	FR693359.1	99	2	MH512865	none
			*Kocuria palustris*		862	MF033107.1	100	1	–	none
Proteobacteria	Alpha	Rhodobacteraceae	*Paracoccus* sp.		808	KJ576895.1	100	2	–	none
	Beta	Comamonadaceae	*Acidovorax* sp.	1	847	KP967489.1	96	1	MH512872	none
			*Acidovorax* sp.	2	839	KP967489.1	98	1	MH512871	none
			*Acidovorax* sp.	3	847	KP967489.1	98	1	MH512870	none
			*Acidovorax* sp.	4	839	KP967489.1	99	1	MH512874	none
			*Acidovorax* sp.	5	855	KP967489.1	99	1	MH512862	none
			*Acidovorax* sp.	6	841	KP967489.1	100	1	–	none
			*Acidovorax* sp.	7	838	KP967499.1	98	1	MH512863	none
			*Acidovorax* sp.	8	844	KP967502.1	97	1	MH512868	none
	Gamma	Aeromonadaceae	*Aeromonas molluscorum*		852	AY532692.1	100	2	–	none
			*Aeromonas media*		852	KP967511.1	99	1	MH512885	none
			*Aeromonas* sp.	1	858	CP017143.1	100	3	–	yes
		Enterobacteriaceae	*Buttiauxella* sp.		849	LN824003.1	100	2	–	none
			*Serratia liquefaciens*		862	MF716555.1	99	1	MH512879	none
		Oceanospirillaceae	*Marinomonas* sp.		846	JF710987.1	100	1	–	none
		Vibrionaceae	*Aliivibrio finisterrensis*		868	HM031457.1	100	2	–	none
			*Enterovibrio calviensis*		852	NR_041741.1	99	2	MH512873	none
			*Enterovibrio nigricans*	1	852	AM942724.1	99	14	MH512886	none
			*Enterovibrio nigricans*	2	852	AM942724.1	99	1	MH512887	none
			*Listonella anguillarum*	1	848	CP011460.1	100	1	–	yes
			*Listonella anguillarum*	2	857	CP023310.1	98	1	MH512880	yes
			*Listonella anguillarum*	3	839	CP023310.1	99	1	MH512867	yes
			*Listonella anguillarum*	4	864	CP023310.1	100	16	–	yes
			*Photobacterium phosphoreum*		859	AY888014.1	99	1	MH512881	yes
			*Photobacterium* sp.	1	837	AB681340.1	99	1	MH512869	yes
			*Photobacterium* sp.	2	859	AB681340.1	99	1	MH512882	yes
			*Photobacterium* sp.	3	855	KC951114.1	96	1	MH512883	yes
			*Photobacterium* sp.	4	852	KC951114.1	100	1	–	yes
			*Vibrio aestuarianus*		864	NR_113780.1	100	2	–	none
			*Vibrio splendidus*		890	JX441440.1	100	1	–	yes
			*Vibrio* sp.	1	866	AB038029.1	99	1	MH512877	none
			*Vibrio* sp.	2	839	EU091323.1	99	1	MH512866	yes
		Moraxellaceae	*Acinetobacter haemolyticus*		849	KC178576.1	99	7	MH512888	none
			*Psychrobacter alimentarius*		833	CP014945.1	100	13	–	none
			*Psychrobacter cibarius*		868	NR_043057.1	100	2	–	none
			*Psychrobacter faecalis*		876	JF710999.1	100	1	–	none
			*Psychrobacter* sp.	1	852	FN377739.1	99	1	MH512856	none
			*Psychrobacter* sp.	2	846	LC184492.1	100	3	–	none
			*Psychrobacter* sp.	3	870	KT583340.1	99	3	MH512861	none
			*Psychrobacter* sp.	4	876	KT583340.1	99	2	MH512864	none
			*Psychrobacter* sp.	5	852	KU364058.1	98	1	MH512860	none
			*Psychrobacter* sp.	6	852	KU364058.1	99	1	MH512859	none
			*Psychrobacter* sp.	7	833	KU364058.1	100	42	–	none
			*Psychrobacter* sp.	8	876	KX621125.1	100	1	–	none
		Shewanellaceae	*Shewanella baltica*		882	AJ000216.1	99	5	MH512878	none
			*Shewanella baltica*		867	CP000753.1	99	1	MH512876	none
			*Shewanella baltica*		862	HM629402.1	99	2	MH512884	none
			*Shewanella baltica*		853	HM629402.1	100	5	–	none
			*Shewanella halifaxensis*		866	NR_074822.1	100	1	–	none
			*Shewanella* sp.	1	866	FR744880.1	100	3	–	none
			*Shewanella* sp.	2	868	JQ765408.1	100	3	–	none
			*Shewanella* sp.	3	853	LK392315.1	100	10	–	none
							∑	195		

Isolates were allocated to the next validly described species or genus (NCBI database) if identity was ≥ 96% and distinction to other closely related species or genera was possible. ^a^ All sequences that revealed an identity < 100% were submitted to NCBI GenBank and accession numbers of the representative isolates are indicated within the table

### Antagonistic activity against pathogens

Thirteen isolates (7.4%) showed an antagonistic activity towards *Tenacibaculum maritimum*. Four of these isolates were members of the *Psychrobacter* genus isolated from wild turbot (Table 2). Seven isolates with antagonistic activity were isolated from farmed turbot and characterized as *Acinetobacter heamolyticus*. Finally, two strains with antagonistic activity closely related to *Enterovibrio calviensis* were isolated from wild European flounder. None of these isolates revealed an antagonistic activity against *Listonella anguillarum* or *Edwardsiella tarda*.

**Table 2:** In vitro characterization of selected candidate probiotics on antagonistic activity in WDAA ^a^, SMA ^b^ and SEFA ^c^

**Isolate** **ID**	**Phylogenetic classification (see Table** **1**)	**Origin**	**Gastro-intestinal segment**	**Association to intestinal surface**	**In vitro characterization**		
					WDAA*	**SMA**	**SEFA**
002	*Psychrobacter* sp. Cluster 2	Turbot, wild *(Scophthalmus maximus)*	stomach	moderate	+		
004	*Psychrobacter* sp. Cluster 7		stomach	moderate		+	+
006	*Psychrobacter alimentarius*		stomach	moderate			+
010	*Psychrobacter alimentarius*		stomach	moderate			+
012	*Psychrobacter* sp. Cluster 2		stomach	moderate		+	++
034	*Psychrobacter* sp. Cluster 7		midgut	strong	+		
047	*Paracoccus* sp.		midgut	moderate			++
054	*Psychrobacter alimentarius*		pyl. Ceaca	moderate			+
060	*Staphylococcus saprophyticus*		midgut	none		+	
062	*Psychrobacter* sp. Cluster 4		stomach	strong		+	
076	*Psychrobacter* sp. Cluster 7		midgut	strong	+		
077	*Psychrobacter* sp. Cluster 7		midgut	strong	+		
094	*Psychrobacter alimentarius*		stomach	strong		+	+
095	*Psychrobacter* sp. Cluster 4		stomach	strong		+	
222	*Acinetobacter haemolyticus*	Turbot, farmed *(Scophthalmus maximus)*	midgut	none	+		
224	*Acinetobacter haemolyticus*		stomach	none	+		
241	*Acinetobacter haemolyticus*		stomach	none	+		
242	*Acinetobacter haemolyticus*		stomach	none	+	+	+
243	*Acinetobacter haemolyticus*		stomach	none	+		
244	*Acinetobacter haemolyticus*		stomach	none	+		
120	*Enterovibrio calviensis*	European flounder, wild (*Platichthys flesus*)	midgut	moderate	+		
121	*Enterovibrio calviensis*		midgut	moderate	+		

^a^ WDAA - well diffusion agar assay ^b^ SMA - metabolization of saponin ^c^ SEFA - synthesis of essential fatty acids * Antagonistic activity towards *Tenacibaculum maritimum*

### Saponin metabolization

Forty-two isolates were selected according to the molecular characterization (one candidate of each taxon identified) and screened for their ability to metabolize saponin as exclusive carbon and energy source. Here, seven isolates (16.7%) were able to metabolize saponin, based on a significant growth observed in the respective medium (Table 2). Among them, six isolates were derived from wild turbot, one was characterized as *Staphylococcus saprophyticus* (060), the other six isolates were assigned to the *Psychrobacter* genus (004 *Psychrobacter* sp., 012 *Psychrobacter* sp., 062 *Psychrobacter* sp., 094 *P. alimentarius*, 095 *Psychrobacter* sp.). Only one isolate, 242 *Acinetobacter haemolyticus*, had been isolated from farmed turbot. Interestingly, in a corresponding solid agar medium, most isolates only revealed vague smears, probably due to saponin leaching to the surface and subsequently increased concentrations. Nevertheless, tiny colonies were often observed (data not shown).

### Fatty acid profile

**Fig. 1:**
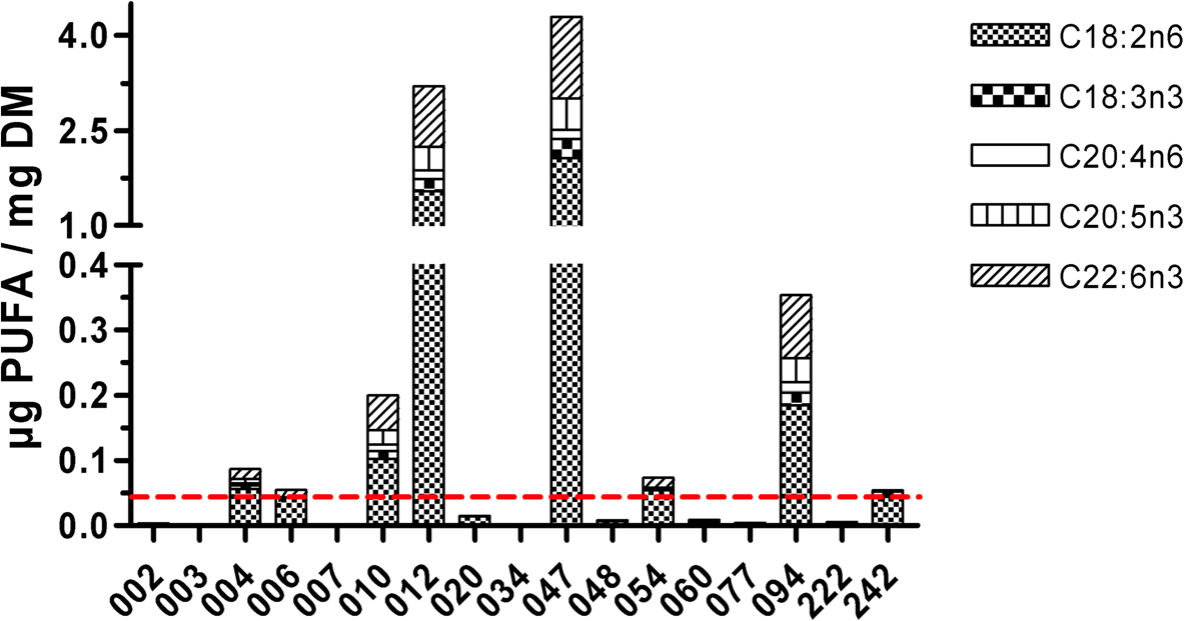
Polyunsaturated fatty acids [μg/mg dry matter] of selected isolates: C18:2(n-6) (LA), C18:3(n-3) (ALA), C20:4(n-6) (ETA), C20:5(n-3) (EPA) & C22:6(n-3) (DHA). Dash line represents median of total PUFA content of all isolates. A three-fold increase of total PUFA was considered relevant for identification of probiotic candidates and indicated as + in Table 2

According to previous results and information on the taxonomic identity of the respective isolate, 17 isolates were selected for the SEFA analysis, focusing on abundant FA as well as those considered particularly interesting (PUFA: ω3 ALA, EPA, DHA, ω6 LA, ETA) as presented in Fig. 1. The variation in the FA profile was remarkably, particularly with regard to the PUFA content. Especially two isolates, 012 *Psychrobacter* sp. and 047 *Paracoccus* sp., revealed a high content of PUFA, in particular DHA, LA and, less significantly, EPA and ALA.

### Viability of probiotics upon diet preparation and storage

**Fig. 2:**
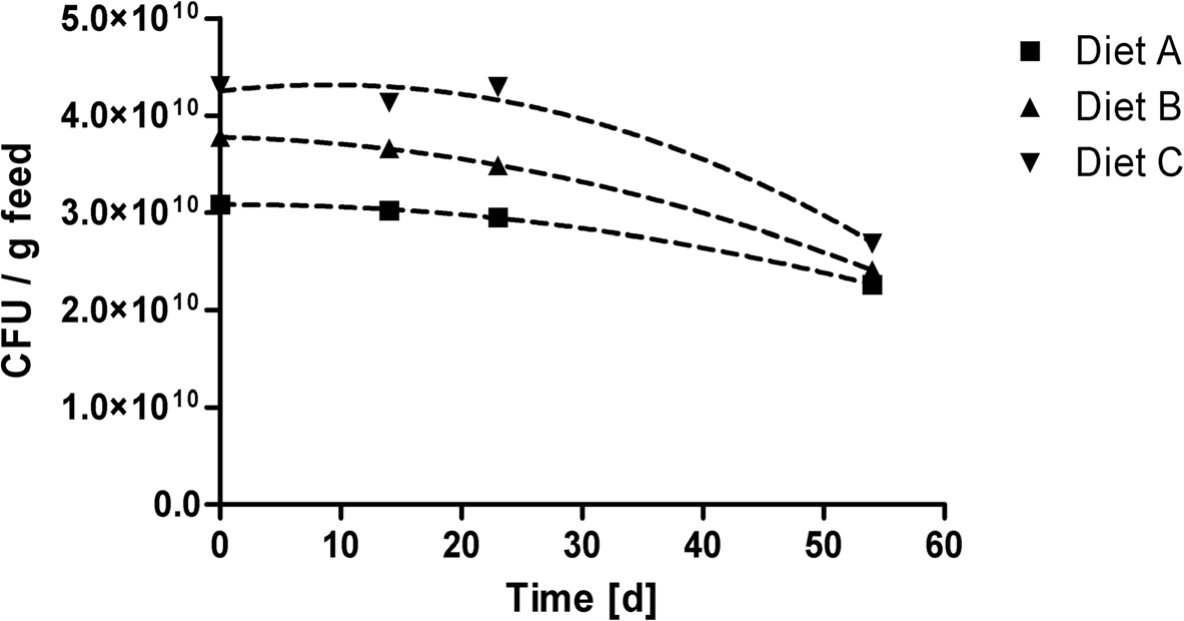
Decrease of viable bacteria in three experimental diets over 54 d of storage after top coating with candidate probiotic suspensions of ■ Diet A (002), ▲ Diet B (077) and ▼ Diet C (242), assessed by plate counting as CFU/g feed. CFU - colony forming units

After coating, vacuum-packed probiotic supplemented feed could be stored at 4 °C for 54 d, without a major decrease in the viability of the respective probiotic isolate monitored as confirmed experimentally in all three diets (Fig. 2). Starting from the day of probiotic supplementation (0 d) to 23 days of storage only a slight decrease of maximum 7.7% (diet B) was observed in all three experimental diets. Still, a comparable decrease of 26.6% (diet A) to 37.6% (diet C) was monitored over time from 0 d to 54 d.

## Discussion

Probiotics have the potential to improve immune status as well as performance of farmed fish but the use of non-native bacteria derived from endothermic terrestrial species may undermine a successful colonization of the GIT of farmed fish. Thus, isolation of native candidates is promising if in vitro screening for beneficial effects allow the extraction of a feasible number of candidates for in vivo evaluation in classical feeding trial or challenge test. Ideally, probiotic candidates should exhibit high growth rates at the respective rearing temperatures, be easily stored in cryo-cultures, oxygen tolerant and effectively supplemented to the diet assuring sufficient viability. Although several demanding strategies such as micro- and bio-encapsulation of the probiotics have been described [46, 47], we present a simple and cost-effective method for the coating that only affected the integrity of the pellets to an acceptable degree.

In the present study several probiotic candidates were identified addressing antagonism towards a major pathogen of turbot, *Tenacibaculum maritimum*, synthesis of essential FA and metabolization of plant-specific anti-nutrients, using saponin as a model substance. Among the autochthonous microbial community closely associated with the tissue surface of the fish intestine, a few pathogens were identified here. This confirms that pathogens are part of the teleost microbiom, even in fish that do not exhibit any symptoms of disease [36, 48]. Molecular characterization may allow the exclusion of pathogenic taxa but in several groups 16S rRNA sequencing does often not provide species-specific identification as observed in this study (Table 2). For example, isolates assigned to *Acinetobacter* and *Psychrobacter* comprise beneficial candidates as well as recorded pathogens, as presented below.

In this study, bacterial isolates were considered as closely associated with the intestinal wall (autochthonous), since faeces were removed and loosely associated, transient bacteria were washed away before detaching candidate bacteria with a non-ionic surfactant (Triton X 100), which is commonly used for this purpose [49, 50]. The ability to attach to the mucosal surface, pH and the resistance to bile acids are particularly important to allow a successful colonization of the intestine [51, 52]. Although there is evidence for a core gut microbiota in teleost fish, the diversity of identified intestinal bacteria is enormous and depending on various biotic and abiotic factors [48, 53]. According to Xing et al. 2013 [36], the predominant bacterial phylum in turbot is Proteobacteria accounting for 93% in 16S rRNA gene sequencing [36]. This is in line with our findings as 89.7% were members of the Proteobacteria (Table 1). Considering that most bacteria in the intestine may not be cultivable with the media and under the conditions used here, there will be a considerable discrepancy to the actual microbiom.

Among the isolates, *Psychrobacter* candidates were particularly interesting due to the fact that *Psychrobacter* species are commonly observed in the aquatic environment as well as commercially important freshwater [54] and marine teleost species, e.g., Atlantic cod *Gadus morhua* [55], mackerel *Scomber scombus* [56], fine flounder *Paralichthys adspersus* [57] or grouper *Epinephelus coioides* [35, 58]. In our study, by far the most frequent genus was *Psychrobacter* accounting for 70 out of 195 (35.9%) isolates based on 16S rRNA information but unfortunately this does not allow species-specific distinction. For future work on specific candidates of this genus molecular characterization will target the gyrB gene [59].

Moreover, *Psychrobacter* has already been evaluated as a probiotic additive in fish nutrition. Lazado et al. reported on antagonistic activity of *Psychrobacter* sp. isolated from the intestine of Atlantic cod against two important fish pathogens *Listonella anguillarum* and *Aeromonas salmonicida* subsp. *salmonicida* [60]. Also, increased activity of digestive enzymes was reported by the same authors, indicating the potential for improved digestion upon feeding probiotic supplements [61]. Similar to the observations here, differential effects on three pathogens were reported. Surprisingly, in all our isolates, no antagonistic effects against two other pathogens frequently reported from turbot culture, *Edwardsiella tarda* and *Listonella anguillarum* were observed. Lazardo et al. demonstrated inhibitory activity against the two pathogens, even when pure supernatant was used [60]. This indicates that *Psychrobacter* strains might be capable of producing and secreting effective antimicrobial substances.

The use as probiotic in fish nutrition was also addressed by a classical feeding trial in the orange-spotted grouper *Epinephelus coioides* [58]*.* Here, *Psychrobacter* sp. as an autochthonous intestinal isolate induced a significantly elevated feed conversion and growth performance, correlated to increased activities of digestive enzymes (e.g., hepatopancreatic protease & lipase, intestinal amylase). Moreover, oxidative stress related parameters like superoxide dismutase were slightly elevated [58]. Congruently, Makled et al. observed increased growth rates and feed utilization as well as immune stimulation (e.g., significantly increased IgM, phagocytic and lysozyme activity) after feeding *Psychrobacter* to Nile tilapia *Orechromis niloticus* [62]. As conclusion, *Psychrobacter* isolates should be investigated in more depth and might be sufficient candidates for future in vivo evaluation in turbot.

One of the most promising isolates of the present culture collection is *Acinetobacter haemolyticus*, revealing strong antagonism towards *T. maritimum*, an interesting profile of essential fatty acids and the ability to metabolize saponin (Table 2). Interestingly, beneficial *Acinetobacter* species have been used as probiotics in shrimp aquaculture [63] but to our knowledge no *Acinetobacter* species have been evaluated as probiotic supplement in fish farming, probably due to increasing reports on opportunistic fish pathogens within this genus [64–67]. However, our isolate assigned to *A. haemolyticus* seems to be taxonomically not related to pathogenic *Acinetobacter* species.

In this study, we found 15.4% (*n* = 30) opportunistic or pathogenic species, which is in line with earlier findings. An uncontrolled proliferation of such species illustrates the outbreak of diseases [68]. Several isolates closely related to common pathogens such as *Vibrio alginolyticus* and *Aeromonas hydrophila* [69] have been suggested as probiotics in former studies [70–72]. Here, *Staphylococcus saprophyticus* 060 revealed saponin metabolization but has previously been suggested as potential pathogen by Yang et al. [35]. Similarly, evidence for the species-specific pathogenicity is often limited and argumentation, thus, strongly based on the phylogenetic relationships.

In aquaculture, particularly in a commercial context, probiotics mostly originate from terrestrial livestock and are frequently members of lactic acid bacteria [46, 73]. However, a successful and thus persistent colonization of these probiotics has never been demonstrated. Native probiotic bacteria are adapted to the ambient environment of the target species and thus, considered more effective, exerting longer lasting beneficial effects once applied [60, 74]. Screening for native isolates should consequently be prioritized in the future. Also, native bacteria can a priori be considered adapted to the environmental conditions in the gut, eliminating the need of further testing as required in non-native probiotics. In vivo screening strategies including assays as the ones used here will support the selection of potential candidates but evaluation in feeding trials limits the number of candidates drastically. The ultimate need for such an experimental evaluation requires any easy and cheap way to formulate experimental diets [74]. Here, we were able to demonstrate viability of probiotic candidates over 54 d upon dietary supplementation.

It is surprising that information on the probiotic diet preparation is often insufficient, including the probiotic cultivation and dosage, details on drying process and shelf life assessment. Here, we demonstrate comparable viability between different probiotic species upon top coating of any formulated diet. Expensive and elaborate probiotic incorporation processes using technologies like drum or vacuum coaters are not required for the preparation and extended storage assures high survival of the probiotic supporting experimental evaluation. After almost eight weeks the viable colony forming counts did not even decrease by a tenth power. When supplementing experimental diets with probiotics for a feeding trial, the need arises to up-scale cell cultivation. Here, bacterial cell should be harvested at the end of the logarithmic growth phase, because a delayed harvest could cause a self-inhibition of bacteria or the production of unwanted secondary metabolites. The potential loss of live and active probionts will undermine evaluation. Therefore, for any evaluation, viability should be assessed experimentally.

Plant derived proteins have been increasingly used as an alternative to fishmeal in aquafeeds [7, 75] but often exhibit deleterious effects on the health and performance of fish [12]. Among those, soybean protein represents a major source of protein for animal nutrition but is particularly challenging for carnivorous finfish due to the adverse effects observed. Although soybean meal has a favorable amino acid composition, substitution rates are kept relatively low to avoid negative effects on growth performance, feed conversion or even severe enteritis, mostly assigned to the secondary metabolite saponin [76]. Therefore, as a model substance to focus on here, we addressed the ability of bacteria to metabolize saponin in a screening test. We identified seven isolates that were able to metabolize saponin as only carbon and energy source at usual concentrations of synthetic nutrient solutions (see SMA). Thereby, it seems plausible that the respective isolates may improve degradation of saponin in the intestine of the host, counteracting detrimental consequences of soybean meal in fish nutrition. In the near future, respective isolates characterized here will be evaluated in a feeding trial.

Next to fishmeal, and currently an even more pressing problem in fish nutrition, is the limitation of fish oil in the context of rapidly increasing aquaculture activities worldwide. Here, its replacement by plant ingredients is more problematic, particularly from a consumer’s perspective, since fish oil is the main source of essential FA such as DHA or EPA. Considering a negligible endogenous synthesis in vertebrates and the outstanding role of these FA for human nutrition, reduced utilization of fish oil severely impacts product quality unless other sources (e.g., genetically modified organisms) assure the supply to the farmed fish. As an alternative, we tried to identify probiotic candidates characterized by high levels of DHA, EPA and the precursors LA, ALA and ETA. Such probiotics upon digestion could provide these essential FA to the farmed fish. The FA profile revealed a remarkable variability between isolates. Highest DHA and EPA levels were observed in the isolates 012 *Psychrobacter* sp. and 047 *Paracoccus* sp. Moreover, both isolates were particularly rich in essential FA precursor LA and ALA (Fig. 1). We consider these candidates highly promising with regard to the supply of essential FA, aiming at an in vivo evaluation.

## Conclusions

When searching for probiotic candidates effective in vitro screening will focus on quantifiable traits that will be translated into beneficial effects in the performance of the farmed species, thereby reducing the number of candidates to be tested. We report the screening of native, autochthonous candidates based on a molecular characterization and subsequent screening for interesting features related to pathogen antagonism, SEFA and metabolization of feed-derived anti-nutrients. The preparation of experimental diets for in vivo evaluation assuring high viability of the candidate is furthermore presented.

## Methods

### Isolation of strains

Potential probiotic bacteria were isolated from the GIT of healthy individuals of wild (North Atlantic) and farmed turbot *Scophthalmus maximus* (Stolt Sea Farm AS, Øyestranda, Norway), wild European flounder *Platichthys flesus* and wild common dab *Limanda limanda* (both Baltic Sea). For sampling, fish were sacrificed upon anesthetization (overdose of 10 mg L-1 tricaine methanesulfonate, MS222) according to the recommendations on humane killing of fish (American Veterinary Medical Association, Canadian Council of Animal Care in Science), the entire GIT of fish was removed, cut open under sterile conditions and divided into five segments: a) stomach, b) pyloric caeca, c) fore-, d) mid-, and e) hindgut (Table 2).

First, intestinal content was removed carefully with a spatula, providing the respective sample of loosely associated bacteria. After rinsing the tissue with a 0.9% (*w*/*v*) saline solution, bacteria strongly associated with the GIT were detached with a detergent solution [0.9% saline with 1% (w/v) Triton X 100] and collected with a pipette after approximately 15 min of incubation. All samples were spread on two non-selective and one selective solid media. Trypticase soy agar supplemented with 2% NaCl (TSA 2%, Carl Roth, Karlsruhe, Germany) was used for the cultivation of halotolerant bacteria and Marine agar 2216 (MA, Difco™, Becton Dickinson, Heidelberg, Germany) for halophilic bacteria. Marine lactic acid bacteria were selectively cultured on De Man, Rogosa and Sharpe agar supplemented with 2% NaCl (Carl Roth, Germany). Agar plates were incubated at 18 °C for two to seven days under aerobic conditions. Morphologically different colonies were picked individually and pure cultures were prepared by streak and re-streaking procedure on fresh media. Pure cultures were stored in duplicates in cryo-vials (Roti®-Store cryo-vials, Carl Roth) at − 80 °C.

### Identification of bacteria

Bacterial isolates were identified by direct sequencing of the partial 16S rRNA gene and BLAST analysis. Therefore, bacterial DNA was extracted from liquid cultures with peqGOLD Bacterial DNA Mini Kit (PeqLab, Erlangen, Germany) followed by PCR amplification with universal eubacteria primers EUB 1F: 5’-AATTGAAGAGTTTGATCATGGCTCA-3′ and EUB 907R: 5’-CCGTCAATTCCTTTRAGTTT-3′ [77] in a final volume of 25 μL [1 × Taq buffer, 1 × TaqMaster PCR enhancer, 1.5 U Taq DNA polymerase (all 5 Prime, Hamburg, Germany), 200 μM of each dNTP (Qiagen, Hilden, Germany), 0.17 μM of each primer (TIB Molbiol, Berlin, Germany), 10–20 ng DNA template] in a Eppendorf Mastercycler Ep Gradient cycler [initial denaturation 4 min at 94 °C, followed by 40 cycles denaturation at 94 °C for 1 min, annealing at 59 °C for 1 min, elongation at 72 °C for 1 min and final extension at 68 °C for 7 min]. To avoid co-amplification of bacterial DNA contamination in the recombinant Taq polymerase, a DNAse I digestion was carried out prior utilization. Here, 2.5 μL of polymerase were mixed with 1 μL 1 × DNAse I (Amplification Grade), 1 μL DNAse I buffer (both Thermo Fisher, Darmstadt, Germany) and sterile diethyl pyrocarbonate water to a final volume of 10 μL. Digestion was carried out for 20 min at room temperature (RT) followed by inactivation at 94 °C for 15 min.

### In vitro screening assays

#### Well diffusion agar assay

A well diffusion agar assay (WDAA) was used to screen liquid cultures of isolates for antagonistic activity towards three common flatfish pathogens: a) *Listonella anguillarum* (ATCC 11323), b) *Edwardsiella tarda* (ATCC 15947) and c) *Tenacibaculum maritimum* (ACC 6.1). Pathogenic bacteria were cultured in 6 mL of the medium used for the respective isolate (2–3 d at 18 °C) until an optical density of OD_600_ 1.5 was reached. For the WDAA, 50 (*L. anguillarum*), 200 (*E. tarda*) or 500 μL (*T. maritimum*) of the respective pathogen culture was added to 12 mL of the melted medium at approximately 41.5–42.5 °C. After solidification and drying (20–25 min), wells were punched into the agar (Ø 3.5 mm) and 25 μL of a 2 d old isolate (approx. 10^7−^ 10^8^ CFU mL^− 1^) grown in marine or TSA 2% broth at 18 °C were added. An antibiotic mix (1:100 dilution, Strep/Amp mix) was used as a positive control. Plates were incubated at 18 °C for one to two days and transparent zones (halos) in the pathogen containing agar were recorded. Isolates were only considered positive when they caused halos of ≥2.0 mm. All isolates characterized as positives were assessed twice to confirm the result.

### Saponin metaboliztation assay (SMA)

For the SMA, isolates were grown in medium that contained saponin as only carbon and energy source [10 g of saponin (pure, extracted from Quillaja bark), 1.0 g NH4Cl, 0.5 g K2HPO4, 0.2 g MgSO4 · 7 H2O, 0.01 mg FeSO4 7 H2O and 0.01 g CaCl2 2 H2O in 1.0 L]. Isolates were incubated in 6 mL at 18 °C for 2–3 d and growth was monitored as OD_600_ compared to a control where isolates were incubated in saponin-free medium. Liquid cultures that revealed more than 0.1 OD_600_ were considered as positive. All isolates characterized as positives were double checked to confirm the result.

### Synthesis of essential fatty acids (SEFA)

For the analysis of the species-specific fatty acid composition, isolates were grown for 24 h at 18°C on the respective liquid medium, pelleted by centrifugation (20 min at 4800×g) and washed twice in sterile 0.9% saline. Approximately 60–75 mg bacterial cell mass (wet weight, WW) was freeze-dried (Alpha 1–4 LOC-1 M, Christ, Osterode, Germany) for 48 h until constant weight of dry matter (DM) was recorded. For SEFA, 15–20 mg DM were homogenized by ultra-sonication for 2 min to break bacterial cell walls and extracted in 15 mL chloroform-methanol (2:1 *v*/v, 3 mg butylated hydroxytoluene as antioxidant) for 3 h on ice under continuous shaking on a horizontal shaker in the dark. After centrifugation (5 min at 9000×g), the upper layer (with the lipids extracted) was transferred to 100 mL flasks and the extraction was repeated twice with 5 mL chloroform-methanol (2:1 *v*/v), for 15 min at RT. The extracts were dried under nitrogen flux and re-dissolved in 2.5 mL methanol. Methylation was carried out in 2.5 mL sulfuric acid methanol solution (2.5%) for 4 h at 80 °C. Fatty acid methyl esters (FAME) were extracted with 4.5 mL hexane after 15 min on ice under continuous shaking and the supernatant was collected after centrifugation (5 min at 9000×g). The extraction was repeated twice with 2 mL hexane. After washing the extracts with 20 mL potassium hydrogen carbonate (280 mg in 100 mL H_2_O dest., pH = 8–9; *w*/*v*), the supernatant was dried under nitrogen flux, dissolved in 200 μL hexane and stored at − 20 °C. FAME were determined with an Agilent 6890 N gas chromatograph (Agilent, USA) equipped with the Agilent 5973 N mass selective detector and a fused silica capillary column (J&W CP-Sil 88 for FAME; Agilent: 100 m × 250 μm × 39 μm). The carrier gas (helium) was set to a constant flow rate of 0.1 mL min^− 1^ using a the following temperature program: initial temperature of the column was 80 °C, held for 1 min, subsequently increased by 4 °C min^− 1^ until 220 °C, kept for 15 min. The temperature of the detector interface was 280 °C. Fatty acid methyl-esters were identified by their retention time and mass spectra in full scan mode (SCAN), in comparison with fatty acid standards (FAME Mix 47,885–4, PUFA n°1–47,033 and PUFA n°3–47,085-4, Supelco, Germany). Signals of specific target ions were used for. Data are presented as absolute concentration (μg g^− 1^ DM). For statistical analyses the quantitative occurrence of highly abundant saturated FAs C14:0, C15:0, C16:0, C17:0 and C18:0 was compared to selected PUFA, in particular ω3 C18:3(n-3) (9,12,15-octadecatrienoic acid, ALA), C20:5(n-3) (5,8,11,14,17-eicosapentaenoic acid, EPA), C22:6(n-3) (4,7,10,13,16,19-docosahexaenoic acid, DHA) and ω6 C18:2(n-6) (9,12-octadecadienoic acid, LA), C20:4(n-6) (5,8,11,14-eicosatetraenoic acid, ETA). Detection and determination limits of the analytical method were 0.1 and 0.4 μg mg^− 1^, respectively. For statistical analysis, values below the quantification limit were set to 0.02 μg mg^− 1^.

### Diet preparation and probiotic viability upon storage

Three isolates 002, 077 (both *Psychrobacter* sp.) and 242 (*Acinetobacter haemolyticus*) (Table 2) were used for the evaluation of the coating procedure. Therefore, 900 g commercial extruded feed pellets (R-3 Europe 22%, Ø 3 mm, Skretting, Norway) were top coated with 300 mL probiotic suspension. This probiotic suspension was prepared from 1 L of liquid culture, harvested at 4600×g for 20 min after 48–72 h of culturing at RT, when an OD600 1.5–2.0 was achieved. Pelleted bacteria were washed twice in sterile 0.9% saline and 2.0 mL cell mass were diluted with 300 mL saline solution. Subsamples of 300 g feed were coated repeatedly with 2 × 50 mL of above probiotic saline suspension including an in-between drying phase. Therefore, feed pellets were distributed into a 1 L wide-neck glass bottle and the suspension was poured centrally onto the pellets. Immediately, the bottle was manually shaken horizontally with rotating movements to coat pellets equally with probiotic solution. Finally, the pellets were spread on plastic tablets under a clean bench. Feed was dried for 30 to 45 min under constant air flow until initial weight was achieved, assuring comparable moisture content. The original structural stability of the feed pellets was conserved during the coating process. The ultimate concentration of colony forming units (CFU) was determined in parallel by plate counting. After final drying pellets were vacuum-packaged into germ-free plastic bags and stored at 4 °C. The viability of probiotic cells was monitored in the supplemented diet by plate counting at 14 d, 23 d and 54 d. Therefore, 1.0 g of the coated feed were incubated in 9.0 mL sterile 0.9% saline for 2 min and gently homogenized with a glass pestle. Serial dilutions were cultured on the respective agar at 20 °C for 48 h in duplicates and CFU were recorded.

## Abbreviations

ALA: 9,12,15-octadecatrienoic acid, C18:3(n-3)
bp: Base pairs
CFU: Colony forming units
DHA: 4,7,10,13,16,19-docosahexaenoic acid, C22:6(n-3)
DM: Dry matter
EPA: 5,8,11,14,17-eicosapentaenoic acid, C20:5(n-3)
ETA: 5,8,11,14-eicosatetraenoic acid, C20:4(n-6)
FA: Fatty acids
FAME: Fatty acid methyl esters
GIT: Gastrointestinal tract
LA: 9,12-octadecadienoic acid, C18:2(n-6)
MA: Marine agar 2216
PUFA: Polyunsaturated fatty acids
RT: Room temperature
SEFA: Synthesis of essential fatty acids
SMA: Saponin metabolization assay
TSA 2%: Trypticase soy agar supplemented with 2% NaCl
WDAA: Well diffusion agar assay
WW: Wet weight
